# Child Injury in Israel: Emergency Room Visits to a Children's Medical Center

**DOI:** 10.1100/tsw.2005.32

**Published:** 2005-03-28

**Authors:** Michal Hemmo-Lotem, Claudia Jinich-Aronowitz, Liri Endy-Findling, Michal Molcho, Michal Klein, Yehezkel Waisman, Yehuda L. Danon, Joav Merrick

**Affiliations:** ^1^ Beterem, National Center for Child Safety and Health, Box 7050, IL-49170 Petach Tiqva, Israel; ^2^ School of Public Health, Haifa University, Haifa, Israel; ^3^ Department of Sociology and Anthropology, Bar Ilan University, Ramat Gan, Israel; ^4^ Department of Emergency Medicine, Schneider Children's Medical Center of Israel, Petach Tiqva and Sackler School of Medicine, Tel Aviv University, Tel Aviv, Israel; ^5^ Barbara and David Kipper Institute of Immunology and Allergy, Schneider Children's Medical Center of Israel, Petach Tiqva and Sackler School of Medicine, Tel Aviv University, Tel Aviv, Israel; ^6^ National Institute of Child Health and Human Development, Faculty of Health Sciences, Ben Gurion University of the Negev, Beer-Sheva and Office of the Medical Director, Division for Mental Retardation, Ministry of Social Affairs, Jerusalem, Israel

**Keywords:** childhood injury, emergency medicine, injury, epidemiology, public health, Israel

## Abstract

The object of this study was to provide data for policy making and prevention program planning in Israel. The study examined all visits to the Department of Emergency Medicine at the Schneider Children's Medical Center in 1996 (41,279 visits in total). Approximately 22.6% of the emergency room patients were admitted following injury. Most (97%) were unintentional injury. Approximately 42% of the patients were less than 4 years old and about 20% were 2 years old. In all age groups, the rate of boys was double. Approximately 92% were Jews. Despite this low rate of non-Jewish patients, however, they constituted 20% of later hospitalizations. The main injuries recorded were bruises and wounds from blunt objects, falls, motor vehicle–related accidents, and sport injuries. The most commonly injured body parts were the head and upper and lower limbs. In 82%, medical treatment was reported and 7% were hospitalized. In examining injuries over the year, there were no significant differences between the different months, but there were clusters of injuries around various holidays—bicycle and skateboard accidents at Rosh Hashanah, Yom Kippur, and Succoth; pedestrian accidents around Lag BaOmer; burns on Purim, Hannukkah, and Passover; and accidental poisoning around Passover. The findings gave an indication of the nature of the injured population groups. These data could be useful for prevention strategy, both on the level of physical injury as well as on the level of the times of the year, when the risk was higher. The data collected very strongly raise the urgent need for establishing a national surveillance system, which would allow tracking injury-related data with respect to young people throughout the country.

## INTRODUCTION

In Israel, as in other developed countries, injuries (accidents or injuries) are the number one cause of death among children and youth aged 1–19 years[1]. Injury also constitutes the primary reason for potential years of life lost up to age 65[2]. Physical injuries also have significant economical consequences, as injuries constitute the principle cause behind short- and long-term disability[2,3,4,5,6]. In addition, children and their families suffer the emotional pain, trauma, and stress. Furthermore, a multinational study among youth showed that the rate of injury among Israeli youth in the community was high compared to other countries[7].

In 1997, as an example, 225 children died as a consequence of injury, while all other causes of death together (infectious diseases, sepsis, pneumonia, birth defects, and more) caused 151 deaths[1]. The most common cause of death from injury was motor vehicle–related accidents of all types (3:100,000), followed by drowning (1:100,000), falls, burns, strangulation, and poisoning (each at a rate of less than 1:100,000)[8]. These rates were similar to the rates found in other countries in the Western world[9,10].

Injury also constitutes a huge factor out of all patients admitted to emergency rooms. During the year 2000, 171,503 children aged 0–17 visited the emergency room following injury, or about one-third of the total number of children and youth visiting the emergency room[1]. The percentage of admittance to the emergency room as a result of injury was lower among children in their first years of life (8.3%), but increased considerably after this age, reaching 42.6% of all admittance among children 5–9 years of age and 53.1% of all admittance among children 10–14 years of age[11]. The highest accident rate was in the 1–4 and 15–17 age groups. Out of all hospitalizations, the rate of 1–4 year olds was the highest, while the rate of 15–17 year olds and babies during their first year of life was the lowest. The number of boys injured was higher than girls in all age groups. Only 3% of visits to emergency rooms were for injuries that were intentional and the rate of intentional injury among those hospitalized stands at 6%[8]. These statistics are particularly serious in light of the great quantity of knowledge amassed in the world regarding injury prevention. Through reasonable and simple means[3,12], 71 to 95% of injuries are preventable and in the U.S., prevention efforts have led to a 35% decline in the rate of death as a result of injury over the last decade[13].

This study presents comprehensive data regarding visits to a tertiary center emergency room following injury with the aim of providing a primary database for policy and prevention program planning.

## METHODS

This study was based on all visits to the Department of Emergency Medicine, Schneider Children's Medical Center in Israel (SCMCI) during 1996, a total of 41,279 visits. The study population did not constitute a representative sample of the young population in Israel, however, due to the absence of a central surveillance system, the importance of which has been discussed in previous studies[10,14]. This population offers a glimpse into injuries among the youth of Israel.

All admissions due to injury were also studied and the following data were extracted from the charts: sociodemographic data (age, sex, religion, place of residence), particulars in connection with the injury (type of accident and the nature thereof, location, seriousness of damage, hospitalization or release), and medical data such as findings from physical examinations, types of tests and imaging, medical consultations held, and treatment recommended on release.

Emergency room visits were sorted according to the different types of injury and comparisons were performed with respect to different types and fields. Where frequency comparison was required, a Chi-square test was performed. Where group comparisons were required regarding continuous variables such as age, t tests or single-factor difference analysis tests were performed, taking into account the existing number of groups. In this article, only findings found to be statistically absolute at a $p < 0.05$ level are presented.

## RESULTS

Out of 41,279 children and adolescents visiting the Department of Emergency Medicine at SCMCI in 1996, 9,309 visits were the result of accidents (22.6%) or, in other words, every fourth patient was the result of an injury. Most of these visits (over 97%) were the result of unintentional injury (accidents); 2.4% were intentional injuries (158), most of which (71.3%) stemmed from violence (112 children) and the minority (18.5%) were admitted following suicidal attempts (29 cases). Out of 9,309 visits, 923 were return visits from children who had previously visited for additional and different injuries, or multiple visits stemming from the same injury.

### Sociodemographic Findings

- **Age —** The findings indicate that 35% of the injured children were under 4 years of age. Among babies and toddlers up to 2 years (26.2%), it was found that injury was more common as the age of the child increased. In this age group, 8.8% were under 6 months old, 1.7% were 7–12 months old, 18.8% were 13–18 months old, and 58.7% were 19–24 months old. Furthermore, 20.3% of all patients in this age group were babies in their second year of life, most in the second half of that year (15.3% of all patients). In this age group, there was a similar rate of boys and girls admitted to the emergency room following injury. From age 3 onwards, the number of patients dropped as the age increased (see Table 1 and Fig. 1). Regarding this age group, it was found that the rate of injury among boys was higher than among the girls, constituting 62.9% of those injured, i.e., a rate of almost 1:2. The injury rate among boys rose with an increase in age (see Fig. 2).
- **Ethnic origin —** 91.6% of patients were Jewish (see Table 1 and Fig. 2). This ratio was very different from the Jewish/non-Jewish ratio in Israel. One cannot conclude that more Jews than Arabs are injured, as this study was not based on a representative sample. On examining the ratio of those later hospitalized, however, it was found that the non-Jewish constituted 20.0% of those hospitalized.
- **Referred from —** About half of those visiting the emergency room (48.1%) came from other medical facilities. Of these, 66.2% were previously examined at community clinics and Health Fund (HMOs) emergency centers, 20.5% by Emergency Medical Services (Magen David Adom), and the rest at schools, private clinics, other hospitals, or Ministry of Health facilities. Most of the children examined before the visit were referred by a general practitioner (GP) (42.4%), 23.9% by pediatricians, 14.7% by medics, and the rest by other specialists. Most of the children who had not been examined prior to arrival came to the emergency room during nighttime hours. On the other hand, about three-quarters of the children who arrived during daytime hours, did so after having been examined by some other health-care worker.
- **Seasonal variations —** The highest rate of injury was during the months of May and June, followed by September, February, and April. No significant differences were observed between the various seasons: 27.9% of injuries occurred during the summer, 27.0% during the spring, 23.2% during the winter, and 22.0% during the autumn (see Fig. 3). In addition to seasonal differences, injury trends during the Jewish holiday seasons were examined. The rates of injury from specific accidents out of all types of injuries during Jewish holidays were analyzed and

**TABLE 1**
**9,309 Visits in 1996 to the Department of Emergency Medicine, Schneider Children's Medical Center in Israel Due to Injury**

| Age | Number of ER Visits | % of All ER Visitors | Girls (%) | Boys (%) | Jewish (%) | Arab (%) |
|---|---|---|---|---|---|---|
| 0 | 548 | 5.9 | 46.4 | 53.6 | 89.2 | 10.8 |
| 1 | 1,883 | 20.3 | 43.1 | 56.9 | 89.2 | 10.8 |
| 2 | 811 | 8.8 | 41.8 | 58.2 | 89.3 | 10.7 |
| 3 | 701 | 7.6 | 39.9 | 60.1 | 88.5 | 11.5 |
| 4 | 617 | 6.7 | 36.5 | 63.5 | 91.1 | 8.9 |
| 5 | 603 | 6.5 | 35.2 | 64.8 | 90.6 | 9.4 |
| 6 | 527 | 5.7 | 35.7 | 64.3 | 92.2 | 7.8 |
| 7 | 519 | 5.6 | 34.1 | 65.9 | 92.2 | 7.8 |
| 8 | 499 | 5.4 | 34.5 | 65.5 | 92.5 | 7.5 |
| 9 | 485 | 5.2 | 35.1 | 64.9 | 93.8 | 6.2 |
| 10 | 507 | 5.5 | 33.8 | 66.2 | 96.2 | 3.8 |
| 11 | 436 | 4.7 | 28 | 72 | 94.7 | 5.3 |
| 12 | 365 | 3.9 | 29 | 71 | 96.4 | 3.6 |
| 13 | 295 | 3.2 | 20.3 | 79.7 | 94.2 | 5.8 |
| 14 | 194 | 2.1 | 25.8 | 74.2 | 93.7 | 6.3 |
| 15 | 164 | 1.8 | 33.5 | 66.5 | 98.8 | 1.2 |
| 16 | 93 | 1 | 40.9 | 59.1 | 95.7 | 4.3 |
| 17+ | 13 | 0.1 | 46.2 | 53.8 | 100 | 0 |
| Total | 9,309 | 100 | 37.1 | 62.9 | 91.6 | 8.4 |

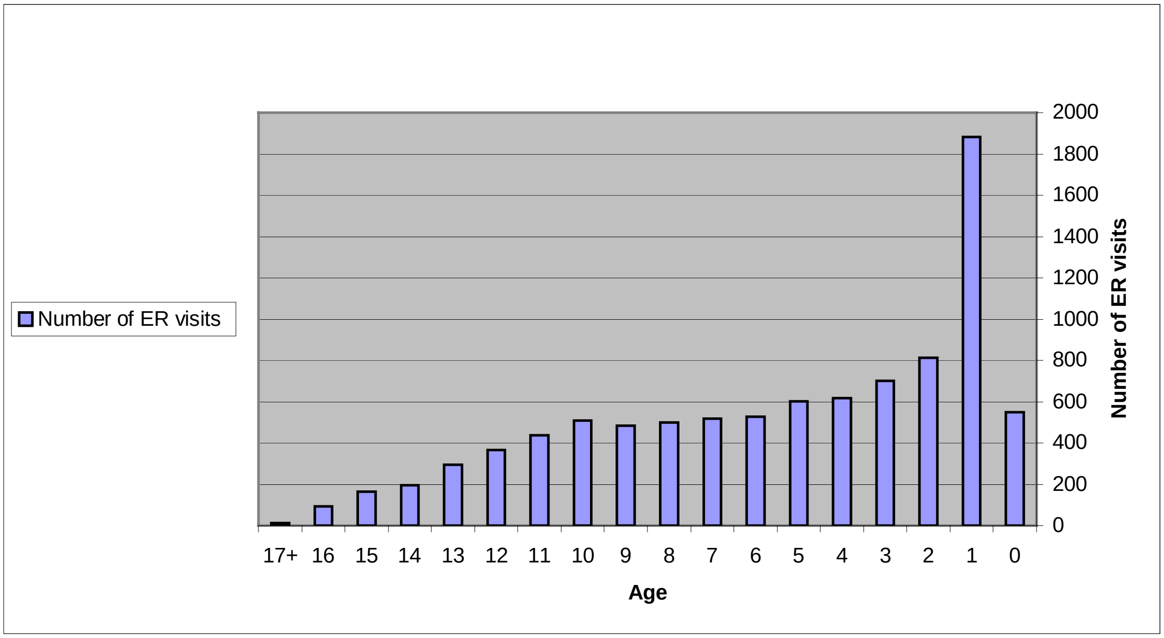

**FIGURE 1**. Number of emergency room visits in 1996 due to injury, by age.

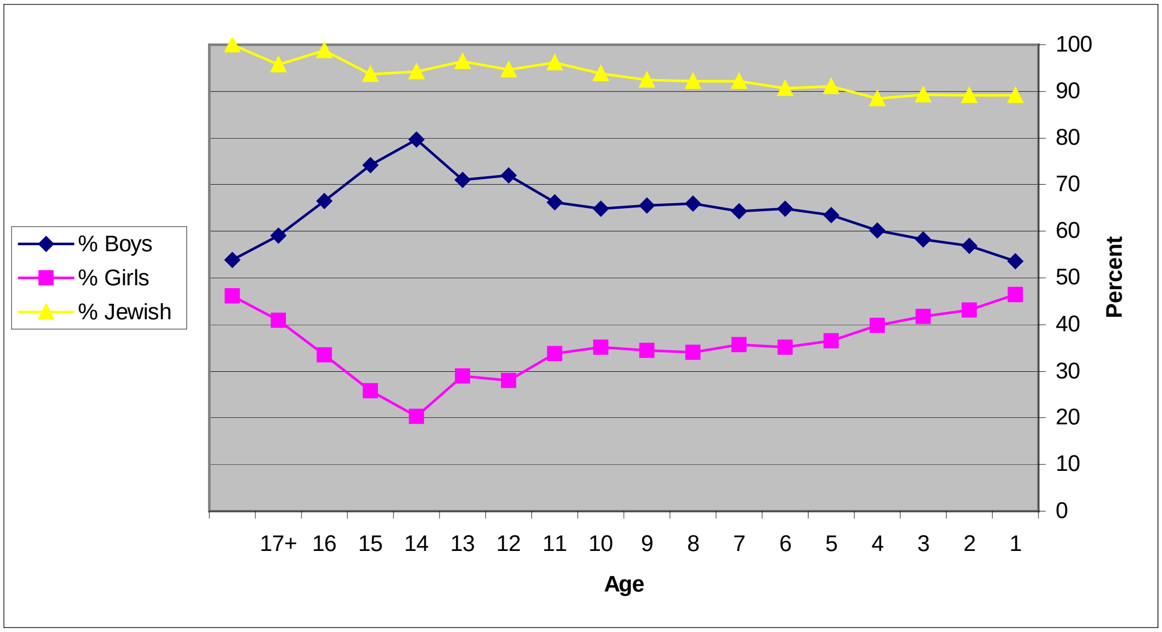

**FIGURE 2.** Emergency room visits due to injury. Percentage by males, females, and Jews out of total emergency room visits.

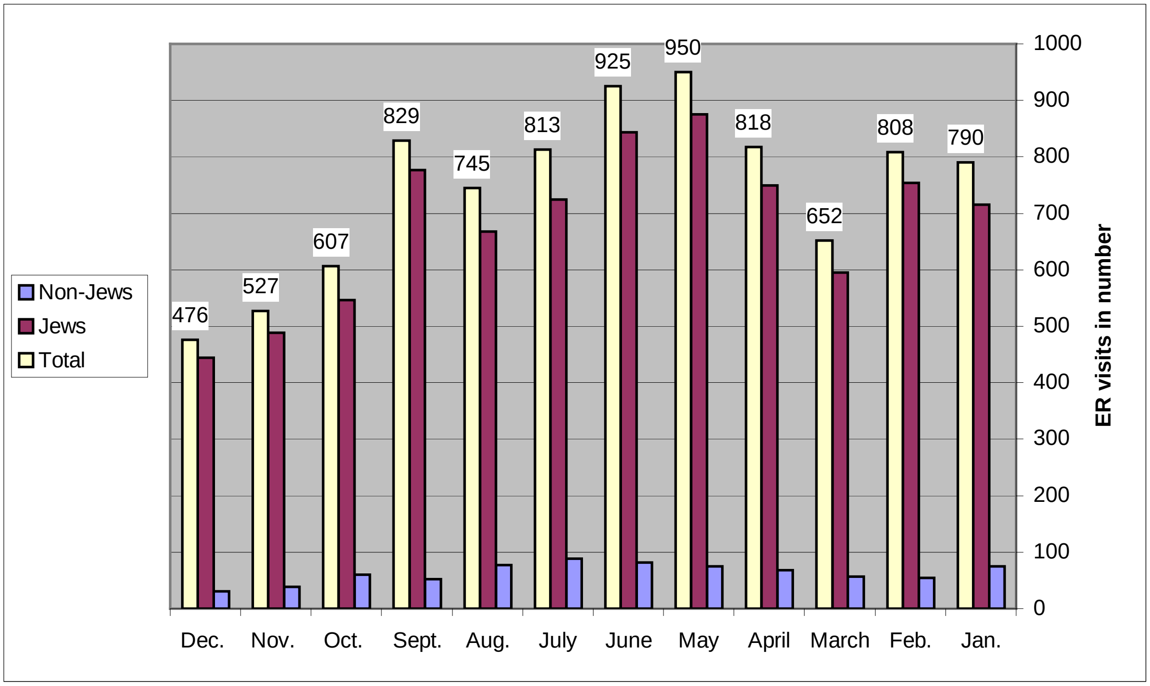

**FIGURE 3.** Seasonal variations, numbers of emergency room visits by month-end, ethnicity.

compared with injuries of the same types throughout the entire year. During Rosh Hashanah, Yom Kippur, and Succoth, bicycle, skateboard, and roller-blade injuries were 2.3, 5.1, and 2.2% higher, respectively, compared with statistics throughout the year. One may observe that the highest increase was on Yom Kippur (20% of injuries on Yom Kippur were these types of injuries as opposed to 3.9% during other times of the year). Pedestrian injuries on Lag BaOmer were 4.3 times higher as opposed to the rest of the year. Burns on Purim, Hannukka, Lag BaOmer, and Passover were 3.5, 3.0, 3.0, and 1.9 times higher, respectively, compared to the rest of the year. Poisoning on Passover was 2.2 times higher than the rest of the year.

- **Cause of injury —** The causes of injury were blunt objects (39.1%); falls (34.1%); motor vehicle–related accidents (7.8%); sport and leisure play injuries (4.2%); penetration of sharp objects into the eye, ear, vagina, and nose (3.7%); burns and electrocution (3.1%); poisoning (2.8%); animal and snake bites (2.2%); swallowing of foreign objects (2.2%); strangulation (0.4%); inhalation of foreign objects (0.2%); and drowning (3 cases, 0.0003%); 1.0% of the cases were not classified. The risk of injury changed with the age of the child. For all ages, bruising injuries from blunt objects and falls were the most common forms of injury. During the first year of life, burns and poisoning were more common. For ages 1–9, falls, motor vehicle–related accidents, and penetration of foreign bodies were the most common injuries and for ages 10–18, the most common were due to falls, sports accidents, and motor vehicle–related accidents (see Table 2).

**TABLE 2**
**9,309 Visits in 1996 to the Department of Emergency Medicine, Schneider Children's Medical Center in Israel Due to Injury (Cause of Injuries in Different Age Groups and Most Frequent Injuries)**

| Age 10–18 Years | Age 1–9 Years | First Year of Life |
|---|---|---|
| Falls | Falls | Falls |
| Injury from blunt objects | Injury from blunt objects | Injury from blunt objects |
| Sports injuries | Motor vehicle–related injuries | Burns |
| Motor vehicle–related injuries | Penetration of foreign bodies | Poisoning |

- **Consequences of injury —** In 68.1% of the cases, clear findings as a result of the injury were documented. From among them: 40.5% suffered from cuts, 35.1% suffered from bodily injury from blunt objects, and 15% of the cases suffered from fractures. In eight cases, the accident required amputation of a limb.
- **The injured organ —** The most common location of an injury was the head; 45.9% of the children arrived with head injuries. The most common were skull injuries (19.6%), forehead (16.0%), eyes (11.2%), chin (9.8%), and nose and lips (6.8% each). Injuries to upper limbs were observed in 25.4% of the children and the most common among these were elbow (17.3%), wrist (16.9%), palm (14.4%), and the lower hand (13.0%). Injuries to the lower limbs were observed in 15.6% and the most common were in the foot (27.2%), ankle (21.4%), and knees (20.9%). A total 5.4% of the injuries were in the trunk and the most common of these were back injuries (26.9%), stomach injuries (21.7%), and chest injuries (21.3%). In 7.7% of the cases, the specific area of the injury was not reported. In 531 cases (6.2%), more than one part of the body was injured. In 1.6% of the cases, there were head and upper limb injuries and in 1.2% of the cases, there were head and body injuries. In nine cases, there were injuries to all parts of the body.
- **Consultations —** In 53.2% of the cases, a specialist was called in for consultation. Out of these, 41.2% of the cases called for an orthopedist; 12.3% for an ear, nose, and throat specialist; 11.6% for an eye specialist; 11.5% for a neurosurgeon; 7.5% for a surgeon; and 6.4% for a plastic surgeon.

- **Treatment —** 82.0% of the cases received medical treatment. The most common treatments were bandaging, cleansing the wound, and stitching. Out of all cases, 193 (2.2%) were asked to remain a few hours for observation and 23 cases required surgery.
- **Hospitalization —** In total, 7.0% were admitted for hospitalization (628 children). The rate of hospitalization following burns and poisons was particularly high, in proportion to the number of emergency room admittance. Most of the children hospitalized (54.0%) were under 4 years of age; 61.7% were sent to the surgical department (385 children), 27.2% to the general pediatric department (170), 20 children were hospitalized in the intensive care unit, 21 in the burns unit, and 10 children in plastic surgery. The rate of hospitalization among non-Jews was much higher than among Jews, 16.0% as opposed to 5.9% accordingly, which constituted 20% of those hospitalized following an injury.

## DISCUSSION

Approximately one-fourth of the children admitted to the Department of Emergency Medicine at Schneider Children's Medical Center in 1996 arrived following an injury. An epidemiological analysis of the injuries and pinpointing groups at risk according to age, sex, socioeconomic status, analysis of events, and systems that lead to such injuries constitute the basis of any plan for prevention strategy and continuous follow-up of environmental and product changes. The cause of these injuries to children differ from case to case, as do the events leading up to the injury. A combination of strategies would, therefore, be the most efficient solution[15]. This study was based on data from one triage hospital located in the center of the country that deals with a large population. The fact that the results of our study were similar to a national study[8] and to data appearing in the annual State of the Child statistics in Israel[11] testifies to the validity of the findings.

Earlier studies that examined the differences in injuries between non-Jewish and Jewish populations in Israel found that non-Jewish children were at higher risk of injury[7] and death, mainly among 0–14 year olds[16]. In this study, we found the number of non-Jews arriving at the emergency room much lower than the number of Jews. This might seem to contradict previous studies. However, as the present study was not based on a representative sample and the hospital where the study was carried out does not treat many non-Jewish areas, one cannot deduce from this that the non-Jews were injured less. Despite the under-representation of non-Jews in this study, it was found that the rate of hospitalization among non-Jewish patients was much higher than the rate among Jewish patients (16% as opposed to 5.9%) and the non-Jews constituted one-fifth of all hospitalizations. This finding supports previous findings where injuries in the non-Jewish sector were more serious than injuries in the Jewish sector. We propose two explanations for this finding. First, it is possible that the injury was more serious as a result of delay in receiving medical attention as a result of both dependence on community medical services as well as the geographical distance of many Arab communities from hospitals. Second, it is possible that their injuries were more serious. From the above, it is clear that we do not have sufficient information to determine the risk factors in the non-Jewish sector and additional studies must be carried out.

Similar to other studies carried out in Israel and throughout the world concerning gender differences, we found that the rate of injury and death among those admitted to the emergency room and hospitalized was higher among boys than girls. This difference was marked already from an early age[8]. The rate of boys involved in accidents arriving at the emergency department in this study was also found to increase with age. Up to the age of 1 year, the boy/girl ratio was almost equal (53% boys). After that, there was a constant increase in the percentage of boys, which reached a peak at age 14, when boys constituted almost 80% of those injured.

There may be a number of explanations for this finding, such as cultural tendencies that encourage boys to be more physical and daring, causing a higher injury rate. Another possibility is that the rate of visits to the emergency room reflects the seriousness of the injury and the rate of injury was similar among boys and girls, but the nature of the injuries among boys was more serious, requiring medical attention at the emergency room, while girls were treated at other medical facilities. There are no current studies with any conclusive findings regarding this question, although this question does arise in almost all studies dealing with the differences in injury between the genders. At the same time, the fact that during the first year of life there is almost no difference between boys and girls strengthens the opinion that the tendency towards injury stems from difference in behavior.

As in similar studies, we found that babies and children aged 1–4 years were at a higher risk of injury[8]. A significant number of injured children were very young; 42% of those injured were babies and toddlers up to age 4 and the highest rate was among 2 year olds. Approximately 20% of all those admitted to the emergency department were children in their second year of life, most in the second half of that year. Most of the children hospitalized were less than 4 years of age. The high rate of injury among young children demands a special emphasis on injury prevention for this age group. In this age group, we found many bruises and injuries from blunt objects, falls, poisoning, and burns. Parents and other care providers must be made aware of small children's abilities, supervision must be increased, and proven tools of prevention provided.

Many children in the emergency department arrived following (in declining order) bruises and injury from blunt objects, falls, and motor vehicle–related injuries. The most common reasons for hospitalization were (in declining order) falls, bruises, and injury from blunt objects, poisoning, and burns. Falls and bruises and injury from blunt objects were the most common reasons for emergency room admittance for all age groups. Among babies in their first years of life, the foremost cause of admittance (after falls and bruises and injury from blunt objects) was burns and poisoning. For children of 1–9 years, the foremost cause (after falls and bruises and injury from blunt objects) were motor vehicle–related accidents and penetration of sharp objects. Children from 10–18 arrived at the emergency room mainly following sports-related accidents and motor vehicle–related accidents. This information must be used in order to provide prevention strategies.

Although no difference was found in the number of emergency room patients, when comparing seasons of the year, there were links between the specific types of injuries and certain holidays. During Rosh Hashanah, Yom Kippur, and Succoth, an increase was observed in the percentage of bicycle, skateboard, and roller-blade accidents. Yom Kippur is a holiday where no cars drive on the roads and children tend to spend the day riding bicycles and roller-blading. Rosh Hashanah and Succoth are just before and after Yom Kippur, and although there are cars on the road, there is an increased use of bicycle and roller-blades on those days also. In light of the large number of injuries, the importance of using means of protection, i.e., helmets and guards, should be emphasized to both parents and children. On Lag BaOmer, there was a noted increase in the percentage of accidents in which children were injured as pedestrians — 4.3 times more than the rest of the year. Lag BaOmer is a holiday where it is customary to light bonfires and on the holiday itself and the days preceding it, young people gather wood for the fire. This collection process is performed in groups on foot and may increase the risk of injury to children as pedestrians. On Purim, Hannukkah, Lag BaOmer, and Passover, an increase was noted in the number of burns (times 3.5, 3.0, 3.0, and 1.9, respectively) in comparison with the rest of the year. This conforms to the holiday customs. On Purim, it is the custom to dress up in costume and there is widespread use of cap guns and caps. The combination of flammable costumes and caps that create sparks increases the risk of burns. Hannukkah and Lag BaOmer are holidays that revolve around fire. On Hannukkah, it is customary to light candles and on Lag BaOmer, bonfires are lit. This proximity to fire raises the risk of burns. On Passover, a large family dinner is held. The proximity of children to cooking pots and stove burners also increases the risk of burns. On Passover, there was also a rise in poisoning from cleaning fluids — 2.2 times higher than the rest of the year. One of the Passover customs is to do spring cleaning in the home. During this cleaning, children's exposure to cleaning fluids increases, thus increasing the chances of this type of poisoning.

Messages with emphasis on burn prevention and keeping cleaning fluids out of the reach of children, storage only in the original bottles, and treatment immediately after intake must be passed on to parents at these holiday times.

Unintentional injury in childhood and youth is a public health concern and there is emerging evidence of the benefits of community-based injury prevention programs[17]. A recent review of ten community-based programs found that effective programs entailed the need for long-term strategy, effective focused leadership, multiagency collaboration, focus on the local community, use of local surveillance, and time to coordinate existing networks and develop new local networks[17].

## CONCLUSIONS

In this study, an overall picture of injuries among children and youth is presented, according to emergency room admittances and hospitalization. The study was performed at Schneider Children's Medical Center located in the center of the country, serving a varied population, Jewish and Arab, city and rural, and a full socioeconomic strata. The results were similar to other studies performed in Israel and the world. The large sample on which this study was based provides a good overall picture and specific division into smaller risk categories, as well as specific causes of injury.

The absence of any exact, consistent, and uniform system for recording injuries prevents an in-depth analysis of all aspects related to the epidemiology of injuries and follow-up of practices over a long-term period. We therefore urge and recommend that a survey system be established throughout the country, for registration and processing of all cases of children admitted to emergency rooms and hospitals following an injury. This, as mentioned, would allow a more in-depth analysis of the data and a deeper knowledge of the general practices and trends, assessment of prevention plans, and appropriate intervention.

## BIOSKETCHES

**Michal Hemmo-Lotem, MD**, is a pediatrician and a graduate of the School of Educational Leadership in Jerusalem. She established Beterem, The National Center for Children's Health and Safety in Petach Tiqva, Israel and is currently the director (CEO). Beterem dedicates its work to childhood injury prevention, safety, and research. Dr. Hemmo-Lotem is the chairman of the National Committee for Childhood Injury Prevention and Safety and a member of the National Council for Child Health and Pediatrics, the National Council for Health Promotion, and is involved in the public debate on childhood injury and safety. She is also a lecturer at the School of Public Health, Haifa University. E-mail: mhemmo@beterem.org

**Claudia J. Aharonovich, RD, MPH,** is the Director of the Out-Reach Department in Beterem, The National Center for Safety and Health in Petach Tiqva, Israel. She is one of the founders of the organization and for the past 9 years has been dedicated to the promotion of safety through working with health and educational professionals at all levels, as well as working with municipalities throughout the country. E-mail: claudiaja@beterem.org

**Liri Endy-Findling, MPH,** was educated at the School of Public Health at the Hebrew University, Jerusalem and is the Director of the Department of Research, Development and Policy at Beterem, the National Center for Children's Health and Safety in Petach Tiqva, Israel. Beterem is an organization focused on injury prevention and safety promotion in childhood and Liri Endy-Findling is one of the leading figures in the organization. She has a rich background as a researcher from her experience at the National Research Institute for Social Sciences and Beterem. She leads public policy, programs development, research management, and promotion of legislation and standards in the field of safety. E-mail: liri@beterem.org.il

**Michal Molcho, PhD,** is a senior research associate in the graduate program of sociology of health, Department of Sociology and Anthropology at Bar Ilan University, Ramat Gan, Israel; a member of the Israeli HBSC (Health Behavior in School-Aged Children) study group; a member of the International HBSC study group; and a member of the Violence and Injury study group within the International HBSC study. E-mail: michal.molcho@nuigalway.ie

**Michal Klein** is the Director of the Information and Development Department at Beterem-Safe Kids in Petach Tiqva, Israel. She is a graduate in communication disorder from the Sackler School of Medicine of the Tel Aviv University and currently a master student at the School of Library, Archive and Information Studies of the Hebrew University, Jerusalem. She specializes in children's safety promotion, injury prevention, resource material, and program development.

**Yehezkel Waisman, MD,** is Director of the Department of Emergency Medicine at the Schneider Children's Medical Center of Israel and senior lecturer in pediatrics at the Sackler School of Medicine, Tel Aviv University. His professional work is devoted to the development of excellence in clinical, educational, academic, and research aspects of pediatric emergency medicine. He established the Pediatric Advanced Life Support (PALS) courses in Israel that subsequently became mandatory for residents in pediatrics and emergency medicine by the Israel Medical Association. Between 1995–99, he was chairman of the Israeli Association for Emergency Medicine and during his term, emergency medicine was recognized as a subspecialty by the Israel Medical Association. Dr. Waisman has numerous publications in the fields of pediatrics, pediatric emergency medicine, and emergency medicine. E-mail: waisy@clalit.org.il

**Yehuda L. Danon, MD,** is a professor of pediatrics and immunology at the Sackler School of Medicine, Tel Aviv University. Formerly Army Surgeon General of the Israel Defense Forces during the Gulf War, Director of the Beilinson Medical Center, Founder of the Schneider Children's Medical Center of Israel, and today the Director of the Kipper Institute of Immunology. Also, Founder and Chairman of the National Council for Child Health and Pediatrics, a think tank on child health advising the Minister of Health. E-mail: ydanon@post.tau.ac.il

**Joav Merrick, MD, DMSc,** is professor of child health and human development affiliated with the Zusman Child Development Center, Division of Pediatrics and Community Health at the Ben Gurion University, Beer-Sheva, Israel; the medical director of the Division for Mental Retardation, Ministry of Social Affairs, Jerusalem; founder and director of the National Institute of Child Health and Human Development. He has numerous publications in the fields of child health and human development, rehabilitation, intellectual disability, disability, health, welfare, abuse, advocacy, quality of life, and prevention. Dr. Merrick received the Peter Sabroe Child Award for outstanding work on behalf of Danish Children in 1985 and the International LEGO-Prize ("The Children's Nobel Prize") for an extraordinary contribution towards improvement in child welfare and well being in 1987. E-mail: jmerrick@internet-zahav.net. Website: www.nichd-israel.com
